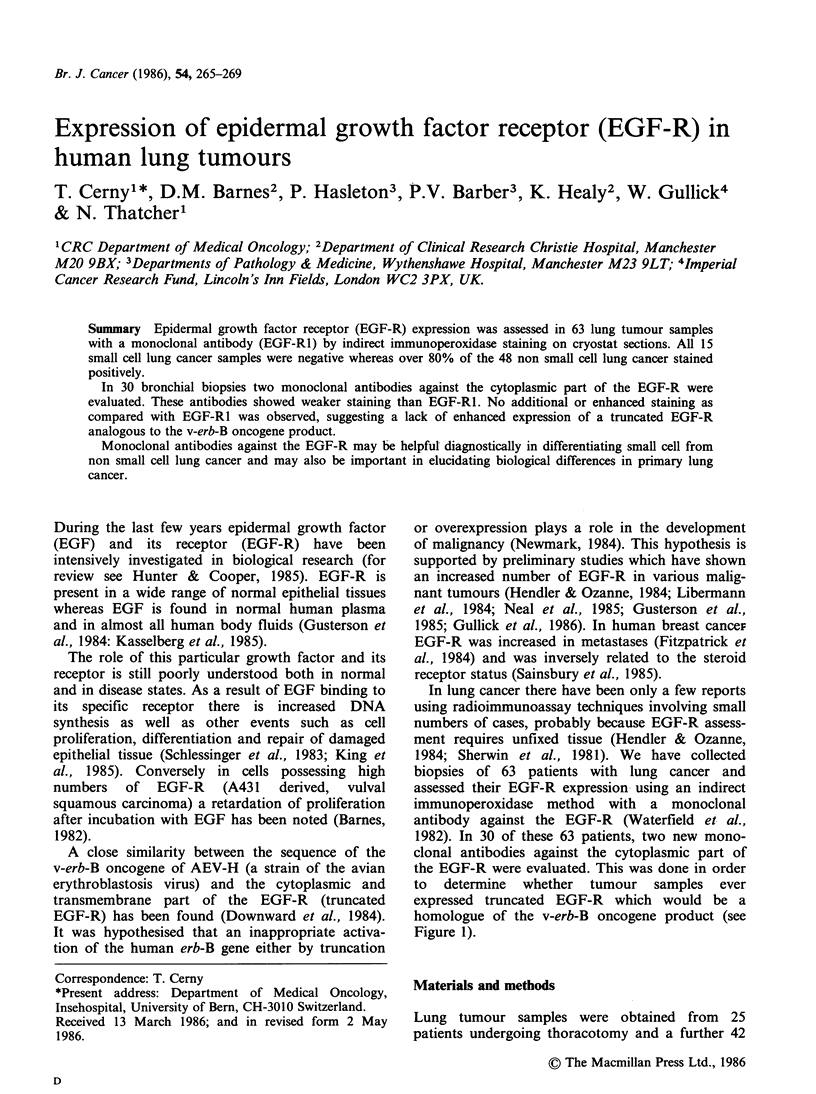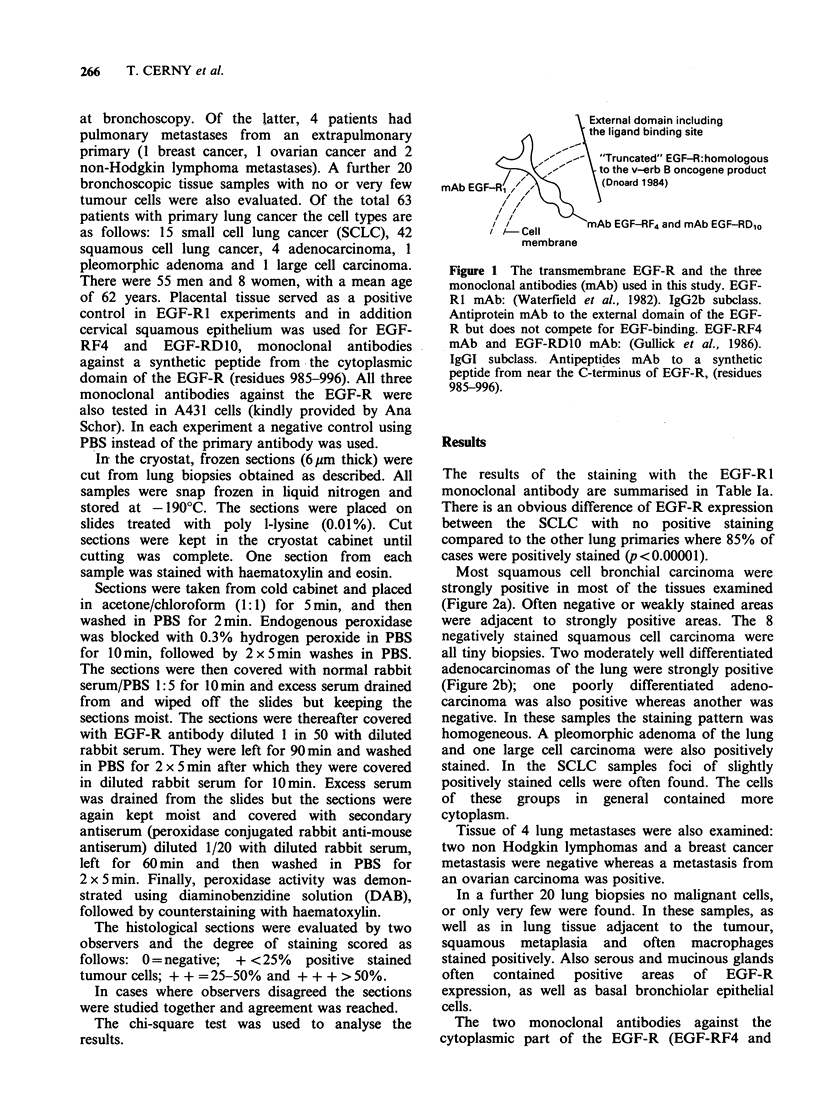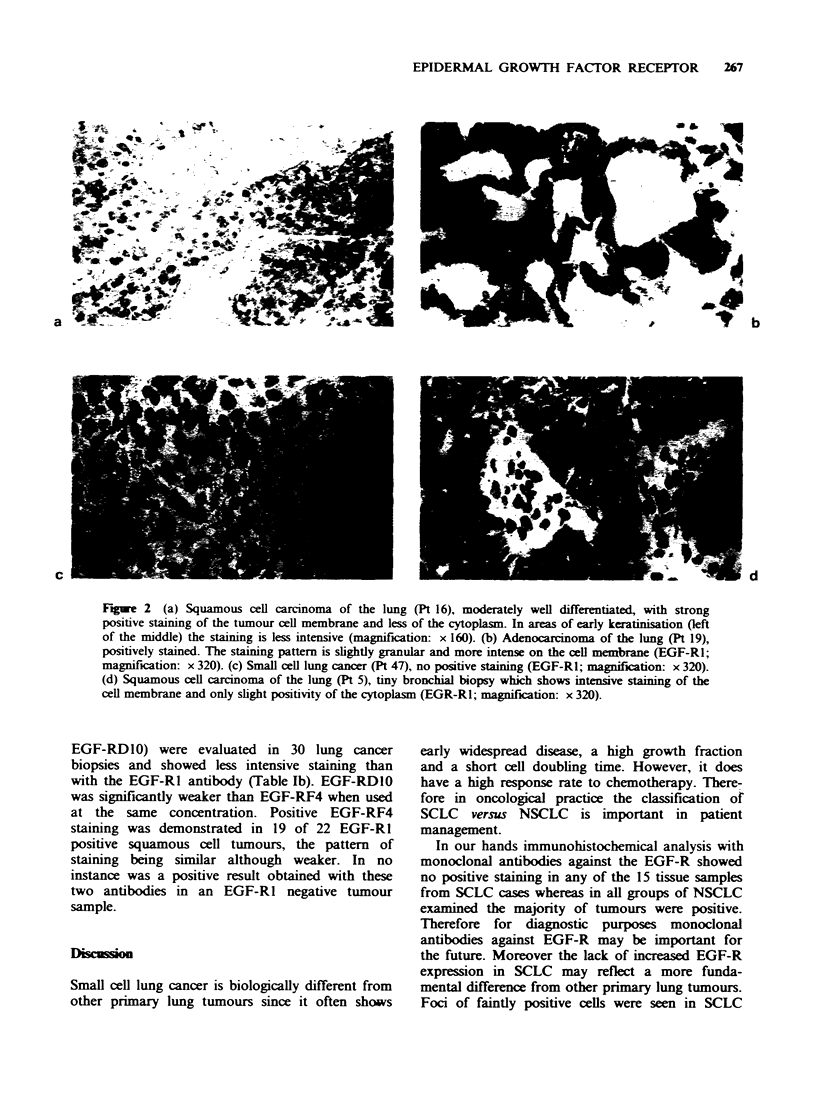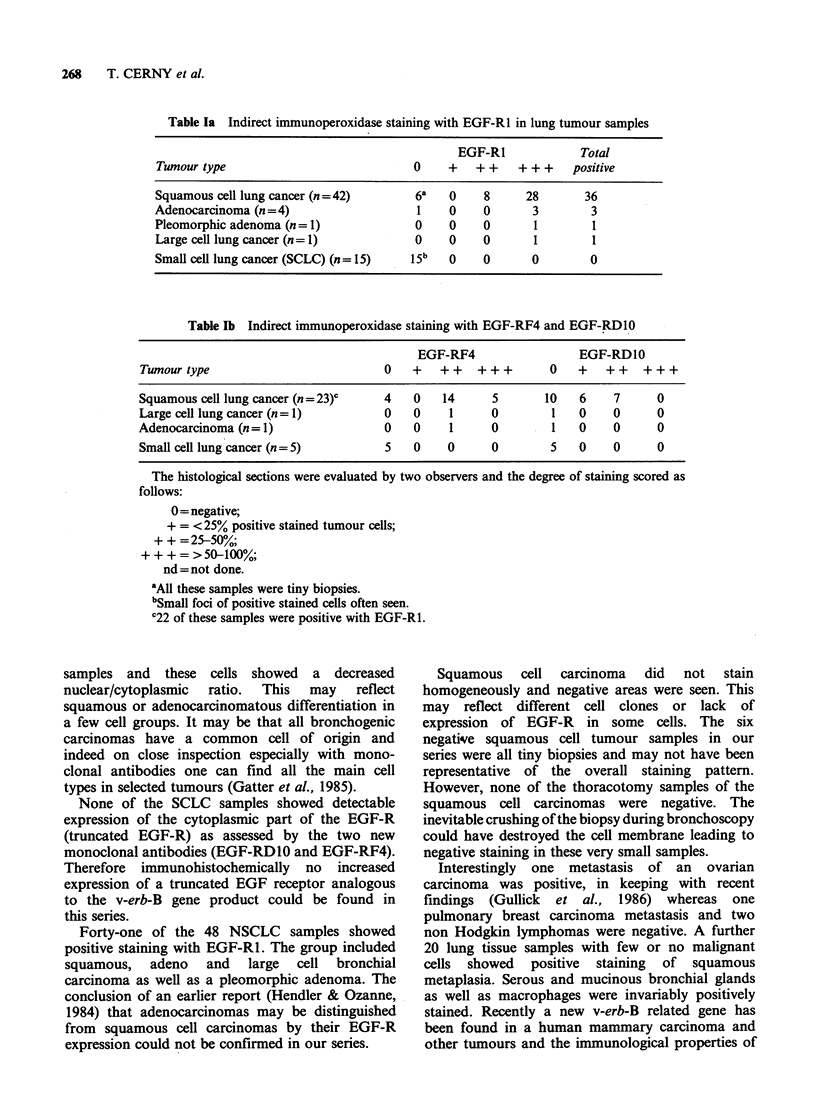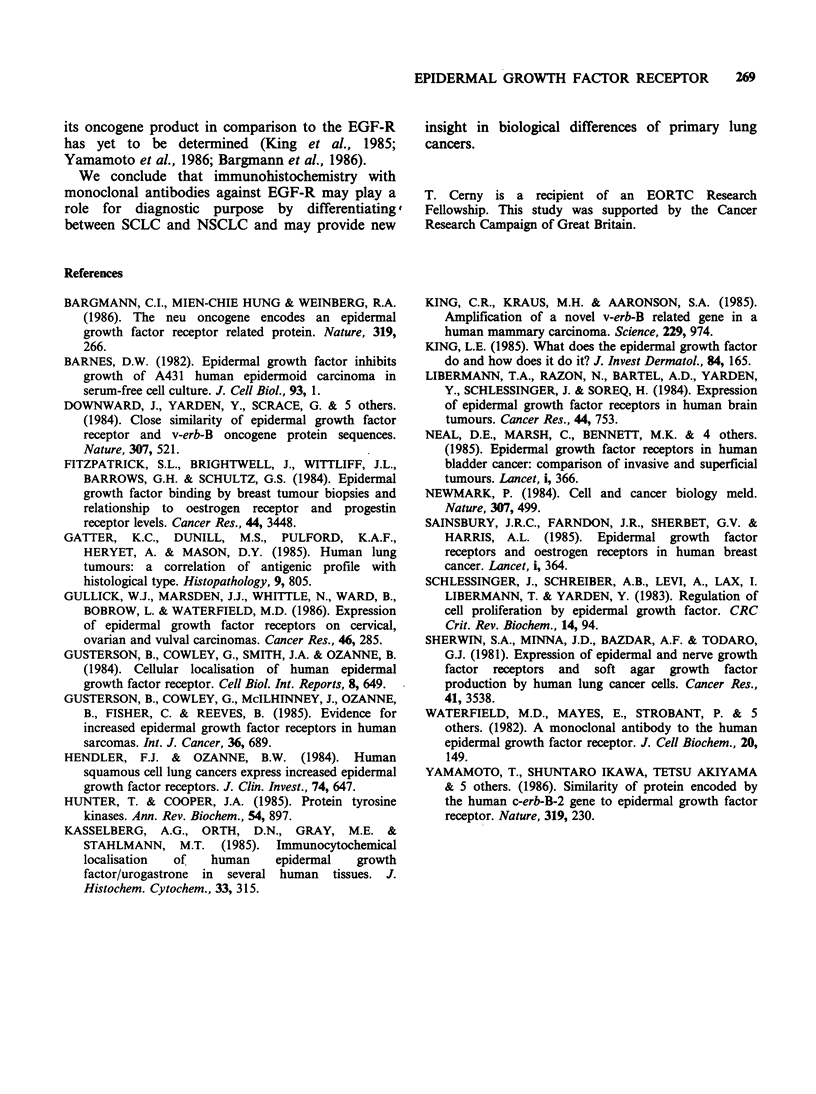# Expression of epidermal growth factor receptor (EGF-R) in human lung tumours.

**DOI:** 10.1038/bjc.1986.172

**Published:** 1986-08

**Authors:** T. Cerny, D. M. Barnes, P. Hasleton, P. V. Barber, K. Healy, W. Gullick, N. Thatcher

## Abstract

**Images:**


*Br. J. Cancer* (1986), **54**, 265–269

### Expression of epidermal growth factor receptor (EGF-R) in human lung tumours

T. Cerny[1]*, D.M. Barnes[2], P. Hasleton[3], P.V. Barber[3], K. Healy[2], W. Gullick[4] & N. Thatcher[1]

[1]*CRC Department of Medical Oncology;* [2]*Department of Clinical Research Christie Hospital, Manchester M20 9BX;* [3]*Departments of Pathology & Medicine, Wythenshawe Hospital, Manchester M23 9LT;* [4]*Imperial Cancer Research Fund, Lincoln's Inn Fields, London WC2 3PX, UK.*

**Summary** Epidermal growth factor receptor (EGF-R) expression was assessed in 63 lung tumour samples with a monoclonal antibody (EGF-R1) by indirect immunoperoxidase staining on cryostat sections. All 15 small cell lung cancer samples were negative whereas over 80% of the 48 non small cell lung cancer stained positively.

In 30 bronchial biopsies two monoclonal antibodies against the cytoplasmic part of the EGF-R were evaluated. These antibodies showed weaker staining than EGF-R1. No additional or enhanced staining as compared with EGF-R1 was observed, suggesting a lack of enhanced expression of a truncated EGF-R analogous to the v-*erb*-B oncogene product.

Monoclonal antibodies against the EGF-R may be helpful diagnostically in differentiating small cell from non small cell lung cancer and may also be important in elucidating biological differences in primary lung cancer.

During the last few years epidermal growth factor (EGF) and its receptor (EGF-R) have been intensively investigated in biological research (for review see Hunter & Cooper, 1985). EGF-R is present in a wide range of normal epithelial tissues whereas EGF is found in normal human plasma and in almost all human body fluids (Gusterson *et al.*, 1984: Kasselberg *et al.*, 1985).

The role of this particular growth factor and its receptor is still poorly understood both in normal and in disease states. As a result of EGF binding to its specific receptor there is increased DNA synthesis as well as other events such as cell proliferation, differentiation and repair of damaged epithelial tissue (Schlessinger *et al.*, 1983; King *et al.*, 1985). Conversely in cells possessing high numbers of EGF-R (A431 derived, vulval squamous carcinoma) a retardation of proliferation after incubation with EGF has been noted (Barnes, 1982).

A close similarity between the sequence of the v-*erb*-B oncogene of AEV-H (a strain of the avian erythroblastosis virus) and the cytoplasmic and transmembrane part of the EGF-R (truncated EGF-R) has been found (Downward *et al.*, 1984). It was hypothesised that an inappropriate activation of the human *erb*-B gene either by truncation or overexpression plays a role in the development of malignancy (Newmark, 1984). This hypothesis is supported by preliminary studies which have shown an increased number of EGF-R in various malignant tumours (Hendler & Ozanne, 1984; Libermann *et al.*, 1984; Neal *et al.*, 1985; Gusterson *et al.*, 1985; Gullick *et al.*, 1986). In human breast cancer EGF-R was increased in metastases (Fitzpatrick *et al.*, 1984) and was inversely related to the steroid receptor status (Sainsbury *et al.*, 1985).

In lung cancer there have been only a few reports using radioimmunoassay techniques involving small numbers of cases, probably because EGF-R assessment requires unfixed tissue (Hendler & Ozanne, 1984; Sherwin *et al.*, 1981). We have collected biopsies of 63 patients with lung cancer and assessed their EGF-R expression using an indirect immunoperoxidase method with a monoclonal antibody against the EGF-R (Waterfield *et al.*, 1982). In 30 of these 63 patients, two new monoclonal antibodies against the cytoplasmic part of the EGF-R were evaluated. This was done in order to determine whether tumour samples ever expressed truncated EGF-R which would be a homologue of the v-*erb*-B oncogene product (see Figure 1).

Correspondence: T. Cerny
*Present address: Department of Medical Oncology, Insehospital, University of Bern, CH-3010 Switzerland.
Received 13 March 1986; and in revised form 2 May 1986.

### Materials and methods

Lung tumour samples were obtained from 25 patients undergoing thoracotomy and a further 42

© The Macmillan Press Ltd., 1986

at bronchoscopy. Of the latter, 4 patients had pulmonary metastases from an extrapulmonary primary (1 breast cancer, 1 ovarian cancer and 2 non-Hodgkin lymphoma metastases). A further 20 bronchoscopic tissue samples with no or very few tumour cells were also evaluated. Of the total 63 patients with primary lung cancer the cell types are as follows: 15 small cell lung cancer (SCLC), 42 squamous cell lung cancer, 4 adenocarcinoma, 1 pleomorphic adenoma and 1 large cell carcinoma. There were 55 men and 8 women, with a mean age of 62 years. Placental tissue served as a positive control in EGF-R1 experiments and in addition cervical squamous epithelium was used for EGF-RF4 and EGF-RD10, monoclonal antibodies against a synthetic peptide from the cytoplasmic domain of the EGF-R (residues 985–996). All three monoclonal antibodies against the EGF-R were also tested in A431 cells (kindly provided by Ana Schor). In each experiment a negative control using PBS instead of the primary antibody was used.

In the cryostat, frozen sections (6 $\mu$m thick) were cut from lung biopsies obtained as described. All samples were snap frozen in liquid nitrogen and stored at $-190°C$. The sections were placed on slides treated with poly l-lysine (0.01%). Cut sections were kept in the cryostat cabinet until cutting was complete. One section from each sample was stained with haematoxylin and eosin.

Sections were taken from cold cabinet and placed in acetone/chloroform (1:1) for 5 min, and then washed in PBS for 2 min. Endogenous peroxidase was blocked with 0.3% hydrogen peroxide in PBS for 10 min, followed by $2 \times 5$ min washes in PBS. The sections were then covered with normal rabbit serum/PBS 1:5 for 10 min and excess serum drained from and wiped off the slides but keeping the sections moist. The sections were thereafter covered with EGF-R antibody diluted 1 in 50 with diluted rabbit serum. They were left for 90 min and washed in PBS for $2 \times 5$ min after which they were covered in diluted rabbit serum for 10 min. Excess serum was drained from the slides but the sections were again kept moist and covered with secondary antiserum (peroxidase conjugated rabbit anti-mouse antiserum) diluted 1/20 with diluted rabbit serum, left for 60 min and then washed in PBS for $2 \times 5$ min. Finally, peroxidase activity was demonstrated using diaminobenzidine solution (DAB), followed by counterstaining with haematoxylin.

The histological sections were evaluated by two observers and the degree of staining scored as follows: 0 = negative; $+ < 25\%$ positive stained tumour cells; $++ = 25$–$50\%$ and $+++ > 50\%$.

In cases where observers disagreed the sections were studied together and agreement was reached.

The chi-square test was used to analyse the results.

**Figure 1** The transmembrane EGF-R and the three monoclonal antibodies (mAb) used in this study. EGF-R1 mAb: (Waterfield et al., 1982). IgG2b subclass. Antiprotein mAb to the external domain of the EGF-R but does not compete for EGF-binding. EGF-RF4 mAb and EGF-RD10 mAb: (Gullick et al., 1986). IgGI subclass. Antipeptides mAb to a synthetic peptide from near the C-terminus of EGF-R, (residues 985–996).

### Results

The results of the staining with the EGF-R1 monoclonal antibody are summarised in Table Ia. There is an obvious difference of EGF-R expression between the SCLC with no positive staining compared to the other lung primaries where 85% of cases were positively stained ($p < 0.00001$).

Most squamous cell bronchial carcinoma were strongly positive in most of the tissues examined (Figure 2a). Often negative or weakly stained areas were adjacent to strongly positive areas. The 8 negatively stained squamous cell carcinoma were all tiny biopsies. Two moderately well differentiated adenocarcinomas of the lung were strongly positive (Figure 2b); one poorly differentiated adenocarcinoma was also positive whereas another was negative. In these samples the staining pattern was homogeneous. A pleomorphic adenoma of the lung and one large cell carcinoma were also positively stained. In the SCLC samples foci of slightly positively stained cells were often found. The cells of these groups in general contained more cytoplasm.

Tissue of 4 lung metastases were also examined: two non Hodgkin lymphomas and a breast cancer metastasis were negative whereas a metastasis from an ovarian carcinoma was positive.

In a further 20 lung biopsies no malignant cells, or only very few were found. In these samples, as well as in lung tissue adjacent to the tumour, squamous metaplasia and often macrophages stained positively. Also serous and mucinous glands often contained positive areas of EGF-R expression, as well as basal bronchiolar epithelial cells.

The two monoclonal antibodies against the cytoplasmic part of the EGF-R (EGF-RF4 and

**Figure 2** (a) Squamous cell carcinoma of the lung (Pt 16), moderately well differentiated, with strong positive staining of the tumour cell membrane and less of the cytoplasm. In areas of early keratinisation (left of the middle) the staining is less intensive (magnification: ×160). (b) Adenocarcinoma of the lung (Pt 19), positively stained. The staining pattern is slightly granular and more intense on the cell membrane (EGF-R1; magnification: ×320). (c) Small cell lung cancer (Pt 47), no positive staining (EGF-R1; magnification: ×320). (d) Squamous cell carcinoma of the lung (Pt 5), tiny bronchial biopsy which shows intensive staining of the cell membrane and only slight positivity of the cytoplasm (EGR-R1; magnification: ×320).

EGF-RD10) were evaluated in 30 lung cancer biopsies and showed less intensive staining than with the EGF-R1 antibody (Table Ib). EGF-RD10 was significantly weaker than EGF-RF4 when used at the same concentration. Positive EGF-RF4 staining was demonstrated in 19 of 22 EGF-R1 positive squamous cell tumours, the pattern of staining being similar although weaker. In no instance was a positive result obtained with these two antibodies in an EGF-R1 negative tumour sample.

### Discussion

Small cell lung cancer is biologically different from other primary lung tumours since it often shows early widespread disease, a high growth fraction and a short cell doubling time. However, it does have a high response rate to chemotherapy. Therefore in oncological practice the classification of SCLC *versus* NSCLC is important in patient management.

In our hands immunohistochemical analysis with monoclonal antibodies against the EGF-R showed no positive staining in any of the 15 tissue samples from SCLC cases whereas in all groups of NSCLC examined the majority of tumours were positive. Therefore for diagnostic purposes monoclonal antibodies against EGF-R may be important for the future. Moreover the lack of increased EGF-R expression in SCLC may reflect a more fundamental difference from other primary lung tumours. Foci of faintly positive cells were seen in SCLC

**Table Ia** Indirect immunoperoxidase staining with EGF-R1 in lung tumour samples

| *Tumour type* | EGF-R1 0 | + | ++ | +++ | *Total positive* |
|---|---|---|---|---|---|
| Squamous cell lung cancer ($n=42$) | 6[a] | 0 | 8 | 28 | 36 |
| Adenocarcinoma ($n=4$) | 1 | 0 | 0 | 3 | 3 |
| Pleomorphic adenoma ($n=1$) | 0 | 0 | 0 | 1 | 1 |
| Large cell lung cancer ($n=1$) | 0 | 0 | 0 | 1 | 1 |
| Small cell lung cancer (SCLC) ($n=15$) | 15[b] | 0 | 0 | 0 | 0 |

**Table Ib** Indirect immunoperoxidase staining with EGF-RF4 and EGF-RD10

| *Tumour type* | EGF-RF4 0 | + | ++ | +++ | EGF-RD10 0 | + | ++ | +++ |
|---|---|---|---|---|---|---|---|---|
| Squamous cell lung cancer ($n=23$)[c] | 4 | 0 | 14 | 5 | 10 | 6 | 7 | 0 |
| Large cell lung cancer ($n=1$) | 0 | 0 | 1 | 0 | 1 | 0 | 0 | 0 |
| Adenocarcinoma ($n=1$) | 0 | 0 | 1 | 0 | 1 | 0 | 0 | 0 |
| Small cell lung cancer ($n=5$) | 5 | 0 | 0 | 0 | 5 | 0 | 0 | 0 |

The histological sections were evaluated by two observers and the degree of staining scored as follows:

0 = negative;
+ = <25% positive stained tumour cells;
++ = 25–50%;
+++ = >50–100%;
nd = not done.

[a]All these samples were tiny biopsies.
[b]Small foci of positive stained cells often seen.
[c]22 of these samples were positive with EGF-R1.

samples and these cells showed a decreased nuclear/cytoplasmic ratio. This may reflect squamous or adenocarcinomatous differentiation in a few cell groups. It may be that all bronchogenic carcinomas have a common cell of origin and indeed on close inspection especially with monoclonal antibodies one can find all the main cell types in selected tumours (Gatter *et al.*, 1985).

None of the SCLC samples showed detectable expression of the cytoplasmic part of the EGF-R (truncated EGF-R) as assessed by the two new monoclonal antibodies (EGF-RD10 and EGF-RF4). Therefore immunohistochemically no increased expression of a truncated EGF receptor analogous to the v-*erb*-B gene product could be found in this series.

Forty-one of the 48 NSCLC samples showed positive staining with EGF-R1. The group included squamous, adeno and large cell bronchial carcinoma as well as a pleomorphic adenoma. The conclusion of an earlier report (Hendler & Ozanne, 1984) that adenocarcinomas may be distinguished from squamous cell carcinomas by their EGF-R expression could not be confirmed in our series.

Squamous cell carcinoma did not stain homogeneously and negative areas were seen. This may reflect different cell clones or lack of expression of EGF-R in some cells. The six negative squamous cell tumour samples in our series were all tiny biopsies and may not have been representative of the overall staining pattern. However, none of the thoracotomy samples of the squamous cell carcinomas were negative. The inevitable crushing of the biopsy during bronchoscopy could have destroyed the cell membrane leading to negative staining in these very small samples.

Interestingly one metastasis of an ovarian carcinoma was positive, in keeping with recent findings (Gullick *et al.*, 1986) whereas one pulmonary breast carcinoma metastasis and two non Hodgkin lymphomas were negative. A further 20 lung tissue samples with few or no malignant cells showed positive staining of squamous metaplasia. Serous and mucinous bronchial glands as well as macrophages were invariably positively stained. Recently a new v-*erb*-B related gene has been found in a human mammary carcinoma and other tumours and the immunological properties of its oncogene product in comparison to the EGF-R has yet to be determined (King *et al.*, 1985; Yamamoto *et al.*, 1986; Bargmann *et al.*, 1986).

We conclude that immunohistochemistry with monoclonal antibodies against EGF-R may play a role for diagnostic purpose by differentiating between SCLC and NSCLC and may provide new insight in biological differences of primary lung cancers.

T. Cerny is a recipient of an EORTC Research Fellowship. This study was supported by the Cancer Research Campaign of Great Britain.

### References

BARGMANN, C.I., MIEN-CHIE HUNG & WEINBERG, R.A. (1986). The neu oncogene encodes an epidermal growth factor receptor related protein. *Nature*, **319**, 266.

BARNES, D.W. (1982). Epidermal growth factor inhibits growth of A431 human epidermoid carcinoma in serum-free cell culture. *J. Cell Biol.*, **93**, 1.

DOWNWARD, J., YARDEN, Y., SCRACE, G. & 5 others. (1984). Close similarity of epidermal growth factor receptor and v-*erb*-B oncogene protein sequences. *Nature*, **307**, 521.

FITZPATRICK, S.L., BRIGHTWELL, J., WITTLIFF, J.L., BARROWS, G.H. & SCHULTZ, G.S. (1984). Epidermal growth factor binding by breast tumour biopsies and relationship to oestrogen receptor and progestin receptor levels. *Cancer Res.*, **44**, 3448.

GATTER, K.C., DUNILL, M.S., PULFORD, K.A.F., HERYET, A. & MASON, D.Y. (1985). Human lung tumours: a correlation of antigenic profile with histological type. *Histopathology*, **9**, 805.

GULLICK, W.J., MARSDEN, J.J., WHITTLE, N., WARD, B., BOBROW, L. & WATERFIELD, M.D. (1986). Expression of epidermal growth factor receptors on cervical, ovarian and vulval carcinomas. *Cancer Res.*, **46**, 285.

GUSTERSON, B., COWLEY, G., SMITH, J.A. & OZANNE, B. (1984). Cellular localisation of human epidermal growth factor receptor. *Cell Biol. Int. Reports*, **8**, 649.

GUSTERSON, B., COWLEY, G., McILHINNEY, J., OZANNE, B., FISHER, C. & REEVES, B. (1985). Evidence for increased epidermal growth factor receptors in human sarcomas. *Int. J. Cancer*, **36**, 689.

HENDLER, F.J. & OZANNE, B.W. (1984). Human squamous cell lung cancers express increased epidermal growth factor receptors. *J. Clin. Invest.*, **74**, 647.

HUNTER, T. & COOPER, J.A. (1985). Protein tyrosine kinases. *Ann. Rev. Biochem.*, **54**, 897.

KASSELBERG, A.G., ORTH, D.N., GRAY, M.E. & STAHLMANN, M.T. (1985). Immunocytochemical localisation of human epidermal growth factor/urogastrone in several human tissues. *J. Histochem. Cytochem.*, **33**, 315.

KING, C.R., KRAUS, M.H. & AARONSON, S.A. (1985). Amplification of a novel v-*erb*-B related gene in a human mammary carcinoma. *Science*, **229**, 974.

KING, L.E. (1985). What does the epidermal growth factor do and how does it do it? *J. Invest Dermatol.*, **84**, 165.

LIBERMANN, T.A., RAZON, N., BARTEL, A.D., YARDEN, Y., SCHLESSINGER, J. & SOREQ, H. (1984). Expression of epidermal growth factor receptors in human brain tumours. *Cancer Res.*, **44**, 753.

NEAL, D.E., MARSH, C., BENNETT, M.K. & 4 others. (1985). Epidermal growth factor receptors in human bladder cancer: comparison of invasive and superficial tumours. *Lancet*, **i**, 366.

NEWMARK, P. (1984). Cell and cancer biology meld. *Nature*, **307**, 499.

SAINSBURY, J.R.C., FARNDON, J.R., SHERBET, G.V. & HARRIS, A.L. (1985). Epidermal growth factor receptors and oestrogen receptors in human breast cancer. *Lancet*, **i**, 364.

SCHLESSINGER, J., SCHREIBER, A.B., LEVI, A., LAX, I. LIBERMANN, T. & YARDEN, Y. (1983). Regulation of cell proliferation by epidermal growth factor. *CRC Crit. Rev. Biochem.*, **14**, 94.

SHERWIN, S.A., MINNA, J.D., BAZDAR, A.F. & TODARO, G.J. (1981). Expression of epidermal and nerve growth factor receptors and soft agar growth factor production by human lung cancer cells. *Cancer Res.*, **41**, 3538.

WATERFIELD, M.D., MAYES, E., STROBANT, P. & 5 others. (1982). A monoclonal antibody to the human epidermal growth factor receptor. *J. Cell Biochem.*, **20**, 149.

YAMAMOTO, T., SHUNTARO IKAWA, TETSU AKIYAMA & 5 others. (1986). Similarity of protein encoded by the human c-*erb*-B-2 gene to epidermal growth factor receptor. *Nature*, **319**, 230.